# Droplet Dynamics of Newtonian and Inelastic Non-Newtonian Fluids in Confinement

**DOI:** 10.3390/mi8020057

**Published:** 2017-02-15

**Authors:** Nikolaos Ioannou, Haihu Liu, Mónica S. N. Oliveira, Yonghao Zhang

**Affiliations:** 1 James Weir Fluids Laboratory, Department of Mechanical & Aerospace Engineering, University of Strathclyde, Glasgow G1 1XJ, UK; nick.ioannou86@gmail.com (N.I.); monica.oliveira@strath.ac.uk (M.S.N.O.); 2 School of Energy and Power Engineering, Xi’an Jiaotong University, Xi’an 710049, China; haihu.liu@xjtu.edu.cn

**Keywords:** droplet dynamics, lattice Boltzmann method, multiphase flows, power–law fluids, droplet deformation, droplet breakup

## Abstract

Microfluidic droplet technology has been developing rapidly. However, precise control of dynamical behaviour of droplets remains a major hurdle for new designs. This study is to understand droplet deformation and breakup under simple shear flow in confined environment as typically found in microfluidic applications. In addition to the Newtonian–Newtonian system, we consider also both a Newtonian droplet in a non-Newtonian matrix fluid and a non-Newtonian droplet in a Newtonian matrix. The lattice Boltzmann method is adopted to systematically investigate droplet deformation and breakup under a broad range of capillary numbers, viscosity ratios of the fluids, and confinement ratios considering shear-thinning and shear-thickening fluids. Confinement is found to enhance deformation, and the maximum deformation occurs at the viscosity ratio of unity. The droplet orients more towards the flow direction with increasing viscosity ratio or confinement ratio. In addition, it is noticed that the wall effect becomes more significant for confinement ratios larger than 0.4. Finally, for the whole range of Newtonian carrier fluids tested, the critical capillary number above which droplet breakup occurs is only slightly affected by the confinement ratio for a viscosity ratio of unity. Upon increasing the confinement ratio, the critical capillary number increases for the viscosity ratios less than unity, but decreases for the viscosity ratios more than unity.

## 1. Introduction

Droplet-based microfluidic technology has recently been exploited to perform microfluidic functions. For example, polymerase chain reaction (PCR) in droplets can avoid PCR inhibition and carryover contamination, which is a serious problem for single-phase microfluidic PCR [1]. However, most available droplet-based microfluidic devices do not have integrated functions and are still far from full automation. One major challenge towards an automated, sample-in and answer-out system is to be able to control dynamical behaviour of droplets on-chip. Although flow speed is usually low, which ensures a laminar flow, droplets are confined in microfluidic channels, and their interfacial dynamics are still difficult to predict. In addition, in microfluidic applications, biological fluids and surfactants are often used, so the fluids can exhibit non-Newtonian rheology. In such microfluidic systems, the flow behaviour of droplets can be very different from Newtonian–Newtonian systems.

The interest in droplet deformation dates back to Taylor [2], with the small deformation $D_{T}$ being described in terms of the viscosity ratio λ (defined as the ratio of the viscosity of the dispersed phase to the continuous phase) and the capillary number Ca as

(1) $$D_{T}=\frac{19\lambda +16}{16\lambda +16}Ca.$$

However, Equation (1) is not able to predict the droplet deformation for very large viscosity ratios [2,3]. Furthermore, Taylor’s theory is for Newtonian fluids and does not take into account effects of shear-dependent viscosity and the influence of the wall proximity [4].

A comprehensive work on a simple shear flow involving shear-thinning fluids (in which the viscosity decreases with increasing shear rates) is reported in [5]. This work examines the deformation and breakup mechanisms of droplets in two experimental setups: (i) Newtonian droplets in a shear-thinning matrix fluid; and (ii) shear-thinning droplets in a Newtonian matrix fluid. The findings show that, in case (i), the deformation of the droplet does not increase linearly with increasing shear rate. However, the orientation angle θ follows the theoretical predictions as reported in [6], regardless of the nature of the suspending fluid, showing a decreasing θ from 35${}^{\circ }$ to 40${}^{\circ }$.

In the case of system (ii), the droplets deform following the Taylor theory for the specific values of apparent viscosity ratio and capillary numbers, indicating that, for a given shear rate, a Newtonian droplet of a specific viscosity can replicate the behaviour of a power–law droplet, which exhibits the same viscosity at that shear rate. Meanwhile, the strongly shear-thinning droplets in a Newtonian fluid exhibit a very weak deformation and high orientation to the flow for small Ca, while noteworthy deformation is observed at a capillary number above Ca≥6 [7]. However, more work is required to understand whether droplets behave differently in a confined microfluidic environment.

Complementing theoretical and experimental studies, numerical simulations have been extensively used to investigate the droplet behaviour in simple shear flow. Numerical simulations give insight into flowfield quantities such as the velocity, pressure and viscosity distributions inside and outside the droplet. In this work, we study droplet dynamics numerically using the lattice Boltzmann method (LBM). The remainder of the paper is organised as follows. First, we describe the numerical method and the simulation setup. In addition to the Newtonian–Newtonian reference case (i.e., a Newtonian droplet in a Newtonian matrix), we also consider the case of a non-Newtonian droplet in a Newtonian matrix, and of a Newtonian droplet in a non-Newtonian matrix. Effects of rheology, geometrical confinement and flow conditions on droplet dynamics are presented and analysed before we conclude the work.

## 2. Simulation Method and Setup

### 2.1. Lattice Boltzmann Method

The lattice Boltzmann method has four major multi-phase models: the colour-gradient model [8], the interparticle-potential model, frequently referred to as the Shan–Chen model [9], the free-energy model [10], and the mean-field theory model [11].

The colour–gradient model, proposed for immiscible fluids [12], integrates a continuum surface force (CSF) model [13] to describe the interfacial tension dynamics. This formulation considers the two phases as a single fluid [14], with space-dependent properties, and replaces the jump condition at the interface with an additional force that acts only in the interface region.

Henceforth, the dispersed fluid is referred to as “red” phase and the continuous as “blue” phase. The respective distribution functions are described as $f_{i}^{R}$ and $f_{i}^{B}$, where the subscript “*i*” denotes the velocity lattice direction and the superscripts “*R*” and “*B*” refer to the red and blue fluids, respectively. Summing the two sets leads to the total distribution function:

(2) $$f_{i}=f_{i}^{R}+f_{i}^{B}.$$

The zeroth moment of each set calculates the density of the respective fluid

(3) $$\begin{array}{cc} \rho^{R} & =\sum_{i}f_{i}^{R},\;\mathrm{red}\;\mathrm{fluid},\\ \rho^{B} & =\sum_{i}f_{i}^{B},\;\mathrm{blue}\;\mathrm{fluid}. \end{array}$$

Moreover, the method utilizes these partial densities to define the colour function or state as the order parameter, which indicates the spatial distribution of the two fluids as

(4) $$\rho^{N}=\frac{\rho^{R}-\rho^{B}}{\rho^{R}+\rho^{B}},\;-1\leq \rho^{N}\leq 1.$$

Thus, the colour function captures an interface of finite thickness. The location where the colour function $\rho^{N}=0$ dictates the interface between the red and blue fluids. Clearly, the total density of the fluid is the sum of the partial densities or the zeroth moment of the total distribution function:

(5) $$\begin{array}{cc} \rho & =\rho^{B}+\rho^{R},\\ \rho & =\sum_{i}f_{i}. \end{array}$$

**Figure 1:**
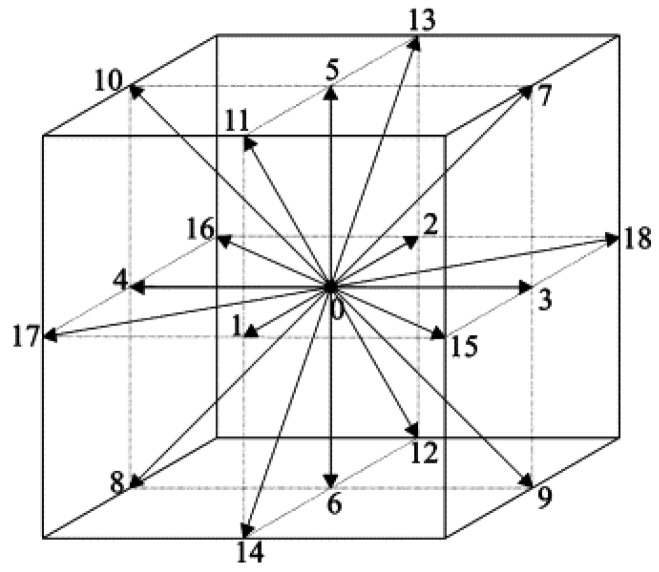
Schematic illustration of the three-dimensional, nineteen velocity (D3Q19) lattice.

In the LBM, the total distribution function ($f_{i}$) usually undergoes a Bhatnagar–Gross–Krook (BGK) collision step as

(6) $$f_{i}^{†}=f_{i}-\frac{1}{\tau }\left(f_{i}-f_{i}^{eq}\right)+\Phi_{i},$$

where $f_{i}$ is the total distribution function in the *i*-th velocity direction at the position *r* and the time *t*, $f_{i}^{eq}$ is the equilibrium distribution function of $f_{i}$, $f_{i}^{†}$ is the post-collision distribution function, *τ* is the dimensionless relaxation time, and $\Phi_{i}$ is the forcing term. The equilibrium distribution function is obtained by a second-order Taylor expansion of Maxwell–Boltzmann distribution with respect to the local velocity $\vec{u}$:

(7) $$f_{i}^{eq}=\rho w_{i}\left[1+\frac{{\vec{e}}_{i}\cdot \vec{u}}{c_{s}^{2}}+\frac{{\left({\vec{e}}_{i}\cdot \vec{u}\right)}^{2}}{2c_{s}^{4}}-\frac{{\vec{u}}^{2}}{2c_{s}^{2}}\right],$$

where $c_{s}$ is the speed of sound, ${\vec{e}}_{i}$ is the lattice velocity in the *i*-th direction, and $w_{i}$ is the weight factor. For the three-dimensional 19-velocity (D3Q19) model, seen in Figure 1, the lattice velocity ${\vec{e}}_{i}$ and the weight factors are given by

(8) $${\vec{e}}_{i}=\left\{\begin{array}{cc} (0,0,0)c, & i=0,\\ (\pm 1,0,0)c,(0,\pm 1,0)c,(0,0,\pm 1)c, & i=1,2,\ldots ,6,\\ (\pm 1,\pm 1,0)c,(0,\pm 1,\pm 1)c,(\pm 1,0,\pm 1)c, & i=7,8,\ldots ,18, \end{array}\right.$$

(9) $$w_{i}=\left\{\begin{array}{cc} 1/3, & i=0,\\ 1/18, & i=1,2,\ldots ,6,\\ 1/36, & i=7,8,\ldots ,18, \end{array}\right.$$

and the speed of sound $c_{s}=\frac{c}{\sqrt{3}}=\frac{\delta_{x}}{\sqrt{3}\delta_{t}}$ with $\delta_{x}$ and $\delta_{t}$ being the lattice length and lattice time step, respectively.

As $\vec{n}=-\frac{\nabla \rho^{N}}{|\nabla \rho^{N}|}$, the interfacial tension force can be written as [13]

(10) $${\vec{F}}_{S}=-\frac{1}{2}\sigma \kappa \nabla \rho^{N},$$

where the local interface curvature *κ* is related to the unit normal to the interface by

(11) $$\kappa =-\left[(I-\vec{n}⊗\vec{n})\cdot \nabla \right]\cdot \vec{n}=-\nabla \cdot \vec{n},$$

and I is the second-order identity tensor. With the interfacial tension force given by Equation (10), the forcing term $\Phi_{i}$ that is applied to realize the interfacial tension effect, reads as in [15]:

(12) $$\Phi_{i}=\left(1-\frac{1}{2\tau }\right)w_{i}\left(\frac{\vec{e_{i}}-\vec{u}}{c_{s}^{2}}+\frac{\vec{e_{i}}\cdot \vec{u}}{c_{s}^{4}}\vec{e_{i}}\right)\cdot {\vec{F}}_{S}\delta_{t}.$$

According to Guo et al. [16], the local fluid velocity should be defined to incorporate the spatially varying interfacial tension force

(13) $$\rho \vec{u}=\sum_{i}f_{i}{\vec{e}}_{i}+\frac{1}{2}{\vec{F}}_{S}\delta_{t}.$$

Using the Chapman–Enskog multiscale expansion, Equation (6) can be reduced to the Navier–Stokes equation in the low frequency, long wavelength limit with the pressure and the fluid viscosity defined by

(14) $$\begin{array}{ccc} p & = & \rho c_{s}^{2}, \end{array}$$

(15) $$\begin{array}{ccc} \eta & = & \rho c_{s}^{2}\left(\tau -\frac{1}{2}\right)\delta_{t}. \end{array}$$

The partial derivatives required for the curvature and normal vector calculations are obtained using the 19-point compact finite difference stencil [17]. To deal with unequal viscosities of the two fluids, the viscosity of the fluid mixture follows a harmonic mean average function to the color function:

(16) $$\frac{1}{\eta \left(\rho^{N}\right)}=\frac{1+\rho^{N}}{2\eta_{R}}+\frac{1-\rho^{N}}{2\eta_{B}},$$

where $\eta_{R}$ and $\eta_{B}$ are the dynamic viscosities of the red and the blue fluids, respectively, as in [17,18,19]. The viscosities $\eta_{R}$ and $\eta_{B}$ are calculated independently from each other, thus each fluid can be Newtonian or power–law.

Although the forcing term generates an interfacial tension, it does not guarantee the immiscibility of both fluids. To promote phase segregation and maintain a reasonable interface, the segregation (recolouring) algorithm of Latva–Kokko and Rothman is used [20]. It can overcome the lattice pinning problem and creates a symmetric distribution of particles around the interface so that unphysical spurious currents can be effectively reduced. The post-segregation (recoloured) distribution functions of the red and blue fluids are

(17) $$\begin{array}{c} f_{i}^{R}=\frac{\rho_{R}}{\rho }f_{i}^{†}+\beta \frac{\rho_{R}\rho_{B}}{\rho }w_{i}\cos\left(\varphi_{i}\right)\left|{\vec{e}}_{i}\right|,\\ f_{i}^{B}=\frac{\rho_{B}}{\rho }f_{i}^{†}-\beta \frac{\rho_{R}\rho_{B}}{\rho }w_{i}\cos\left(\varphi_{i}\right)\left|{\vec{e}}_{i}\right|, \end{array}$$

where β is the segregation parameter and is set to be 0.7 for numerical stability and model accuracy [21]; and $\varphi_{i}$ is the angle between the colour gradient and the lattice vector ${\vec{e}}_{i}$, which is defined by

(18) $$\cos\left(\varphi_{i}\right)=\frac{{\vec{e}}_{i}\cdot \nabla \rho^{N}}{|{\vec{e}}_{i}||\nabla \rho^{N}|}.$$

After the recolouring step, the red and blue distribution functions propagate to the neighbouring lattice nodes, known as propagation or the streaming step:

(19) $$f_{i}^{j}\left(R+\vec{e_{i}}\delta_{t},t+\delta_{t}\right)=f_{i}^{j},\;j=R\;\mathrm{or}\;B,$$

and the resulting distribution functions are then used to calculate the densities of both fluids as in Equation (3).

### 2.2. Geometry, Mesh and Boundary Conditions

**Figure 2:**
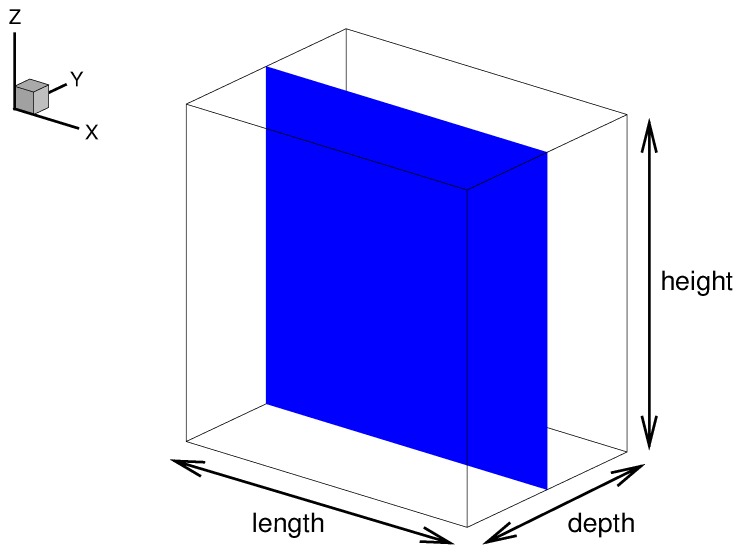
Simulation geometry. The blue plane illustrates the “*x*-*z* middle plane” described in the text.

The computational domains are simple three-dimensional cuboids on the Cartesian coordinate system. The length, depth and height of a domain are assigned to the *x*-, *y*- and *z*-directions, respectively. Each domain is meshed by structured orthogonal grids and the D3Q19 lattice is implemented. A cross sectional slice in the *x*-*z* plane positioned in the middle of the *y*-axis, shown blue in Figure 2, will be referred to as the “*x*-*z* middle plane”, or plainly “middle plane”. In this characteristic plane, the vector and tensor properties are nullified in the *y*-direction due to symmetry. The periodic boundary condition is applied at the inlet and outlet normal to *x*- and *y*-, and the half-way bounce-back at the solid walls in the *z*-direction.

Grid independence tests in a typical shear test showed a variation in the deformation parameter up to 4.5% for a droplet with radius *R* = 10 lattice units (l.u.), and below 1% for a droplet of 30 l.u. The droplet is considered sufficiently isolated for length and depth of 100 and 71 l.u., respectively. However, in high intensity shearing, i.e., high capillary number cases, a droplet can elongate significantly, so the length of the domain will increase to 492 l.u. The height *H* varies within a range of 51 to 223 l.u. to obtain different confinement ratios from 2R/H=0.18 to 0.8.

### 2.3. Initial Conditions and Fluid Properties

In all the simulations, the initial droplet (red fluid) is in the centre of the domain, while the matrix fluid consists only of blue fluid. The velocity everywhere in the domain is $\vec{u}=\left(0,0,0\right)$. At the first time step, the velocities on the top and bottom walls are set to ${\vec{u}}_{t}=\left(U_{w},0,0\right)$ and ${\vec{u}}_{b}=\left(-U_{w},0,0\right)$, respectively. The magnitude of the velocity at the walls ($U_{w}$) and the viscosity of the continuous fluid ($\eta_{c}$) are imposed considering the Reynolds (Re) and the capillary (Ca) numbers, along with the height of the domain.

Non-Newtonian fluids are modelled using a power–law constitutive equation

(20) $$\eta \left(\left|\dot{\gamma }\right|\right)=k{\left(\left|\dot{\gamma }\right|\right)}^{n-1},$$

where *k* is the consistency index, *n* the power law index and $\dot{\gamma }$ the shear rate. For n=1, the viscosity is independent of the shear rate and the fluid is Newtonian; for n<1, the fluid exhibits a shear thinning behaviour, with the viscosity decreasing with increasing shear rate; on the other hand, when n>1, the fluid is shear-thickening, with the viscosity increasing with increasing shear rate.

The Re and Ca numbers are defined using the properties of the continuous-matrix fluid as

(21) $$Re=\frac{\rho {\dot{\gamma }}_{0}R^{2}}{\eta_{c}},\;Ca=\frac{{\dot{\gamma }}_{0}R\eta_{c}}{\sigma },$$

where the *R* refers to the radius of the droplets and σ is the interfacial tension. The shear rate can therefore be written as

(22) $${\dot{\gamma }}_{0}=\sqrt{ReCa\frac{\sigma }{\rho R^{3}}},$$

and the velocity magnitude on the walls ($U_{w}$) is calculated from

(23) $${\dot{\gamma }}_{0}=\frac{U_{w}}{H/2}\Leftrightarrow U_{w}=\frac{{\dot{\gamma }}_{0}H}{2}.$$

For the range of parameters used, the imposed velocity is low enough to neglect compressibility of fluids [22]. Even for a large capillary number, e.g., Ca=1, the Mach number is less than 0.01. Finally, the viscosity of the continuous-matrix fluid ($\eta_{c}$) can be written as

(24) $$\eta_{c}=\sqrt{\frac{Ca}{Re}\rho \sigma R},$$

and the viscosity ratio is

(25) $$\lambda =\frac{\eta_{d}}{\eta_{c}}$$

when a non-Newtonian matrix fluid is used, and the viscosity depends on the shear rate. A set of modified dimensionless numbers that account for this dependency become

(26) $$Re=\frac{\rho {\dot{\gamma }}_{0}^{2-n_{c}}R^{2}}{k_{c}};\;Ca=\frac{{\dot{\gamma }}_{0}R\eta_{c}}{\sigma }=\frac{{\dot{\gamma }}_{0}R\left(k_{c}{\dot{\gamma }}_{0}^{n_{c}-1}\right)}{\sigma },$$

using the shear rate dependent viscosity and the droplet radius as characteristic properties. Clearly, when the exponent is unity i.e., $n_{c}=1$, the fluid is Newtonian and Equation (26) reduces to Equation (21).

In the simulations, the consistency index i.e., *k*, is used to define power–law viscosity ratio $\lambda_{PL}$. For example, the viscosity of the Newtonian droplet ($\eta_{d}$) equals the consistency index of the matrix fluid ($k_{c}$) to obtain a unity viscosity ratio. Finally, note that the interfacial tension is considered as a free parameter and is set to σ=0.001 to minimize spurious currents.

## 3. Results and Discussion

### 3.1. Newtonian Droplet in a Non-Newtonian Matrix Fluid under Simple Shear

**Figure 3:**
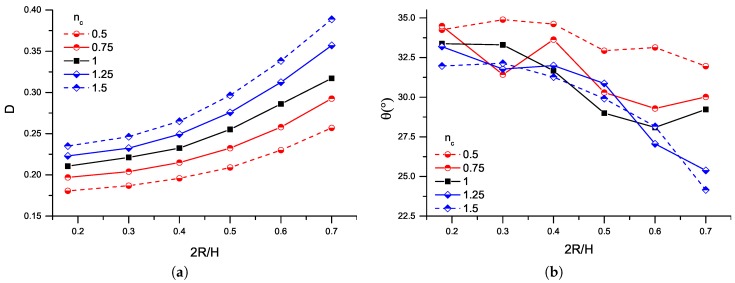
(**a**) deformation parameter; and (**b**) orientation angle as a function of the confinement ratio, 2R/H, for a Newtonian droplet in a range of matrix fluids (Newtonian and power–law fluids, with power–law index $n_{c}$) at Ca=0.2 and λ=1.

First, we examine the confinement effect while keeping the capillary number fixed at Ca=0.2. Figure 3 shows the variation of the deformation parameter and the orientation angle as a function of the confinement 2R/H. For all the cases studied, increasing confinement leads to more deformed droplets that are more oriented towards the shear flow. Additionally, the droplet deforms to a greater extent in the carrier fluid that exhibits a high power–law index, i.e., the drop deforms more in a shear-thickening carrier fluid ($n_{c}>1$) than in a Newtonian carrier fluid ($n_{c}=1$), and deforms less in shear-thinning ($n_{c}<1$) carrier fluid. The more confined the droplet is, the higher the shear stress exerted on its surface. Additionally, shear stress becomes larger as the power–law index increases. This relative increase in the stresses can explain the behaviour of the deformation curves in Figure 3.

Furthermore, the velocity fields (not shown here) are similar to the equi-viscous Newtonian case [4]. The difference is that the high pressure point shifts further from the droplet surface and slightly closer to the centreline when the matrix fluid is more shear thickening, while the exact opposite happens when the carrier fluid is shear-thinning.

We have also investigated the dynamics of droplet breakup and the critical capillary number for droplet breakup under confinement ratios ranging from 0.37 to 0.8. Although the LBM has been used extensively to simulate the droplet breakup [23,24,25,26], to the best of our knowledge, no studies cover such a broad range of parameters. This is partially because of the unsolved technical issues, such as the artificially enlarged interface thickness and case-dependent mobility in the phase-field-based model, high spurious currents as well as the numerical instability in the interparticle-potential model. The color-gradient model we adopt here allows us to be able to access a high viscosity ratio, which is essential for dealing with power–law fluids and low capillary number [21,27].

**Figure 4:**
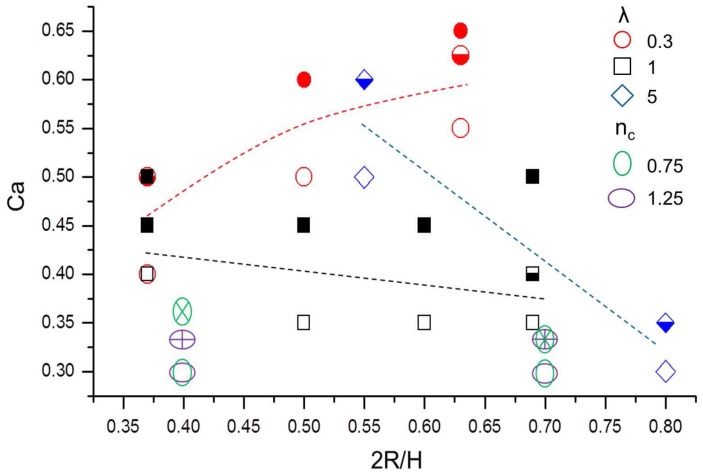
The effect of capillary numbers and confinement ratio on the droplet steady deformation or breakup for the case of a Newtonian droplet in Newtonian matrix fluids (λ=0.3,1,5 and $n_{c}=1$), and in power–law matrix fluids ($n_{c}=0.75,1.25$ and $\lambda_{PL}=1$). The open symbols depict steady states. The half-filled symbols depict binary breakup, and the full symbols ternary breakup for the Newtonian cases, while the crossed symbols show binary breakup for droplets in power–law matrix fluid. The lines highlight the critical capillary numbers for the case of the Newtonian fluid.

In Figure 4, we consider a Newtonian carrier fluid ($n_{c}=1$) with three typical viscosity ratios, i.e., λ=0.3, 1 and 5, and two power–law matrix fluids ($n_{c}=0.75$ and 1.25) for a single viscosity ratio ($\lambda_{PL}=1$). Figure 4 shows a map of drop configuration in the capillary number and confinement ratio parameter space. For the Newtonian fluid, empty symbols represent the steady state regime, while half-filled and full-filled symbols distinguish the binary and ternary breakup modes. The same graph includes two cases of breakup in a power–law matrix fluid. The green ellipses show the cases for $n_{c}=0.75$ and the purple ones for $n_{c}=1.25$. Empty symbols again represent the steady state regime, while crossed symbols denote binary breakup. The critical capillary number ($Ca_{cr}$) for drop breakup lies between the empty and filled or crossed symbols and, in the case of the Newtonian fluid, is indicated with the dashed lines.

For the Newtonian carrier fluid, we observe that the effect of confinement ratio on the critical capillary number depends on the viscosity ratio. For the low viscosity ratio, i.e., λ = 0.3, the critical capillary number varies from $0.4\leq Ca_{cr}\leq 0.5$ at 2R/H=0.37, to $0.5\leq Ca_{cr}\leq 0.6$ at 2R/H=0.5 and finally to $0.55\leq Ca_{cr}\leq 0.625$ at 2R/H=0.625, suggesting that $Ca_{cr}$ increases with increasing confinement.

Furthermore, Figure 4 shows that the confinement ratio has only a small effect on the critical capillary number, which is around 0.4. These findings agree well with the previous experimental and numerical studies [28,29]. It should be noted that, for λ=1, the critical capillary number is known to exhibit a minimum critical capillary number occurring at 2R/H=0.5 [4].

Even though the overall agreement is quite satisfactory between the present results and those obtained by Janssen et al. [28], some quantitative differences are also noticed. For example, Janssen et al. [28] finds experimentally that binary breakup occurs for a unit viscosity ratio droplet at Ca=0.45 and 0.37≤2R/H≤0.45, where our simulations produce a ternary breakup. In addition, the previous experimental study indicates that the lowest $Ca_{cr}$ for all of viscosity ratios (0.3≤λ≤5) and confinement ratios (0.1≤2R/H≤0.9) is around 0.4 [28]. However, in our simulations with a Newtonian fluid matrix, this value is slightly lower (0.35), clearly seen in Figure 4.

Similar to the Newtonian case, when the droplets are immersed in a shear-thinning or thickening fluid, the critical capillary number does not seem to vary significantly with confinement ratio for $\lambda_{PL}=1$, despite it being smaller than that in the Newtonian case.

### 3.2. Non-Newtonian Droplets under Shear

For droplets made of power–law fluid, they are now referred to as “power–law droplets”. The viscosity, consistency index and the exponent of a power–law droplet are noted with $\eta_{d}$, $k_{d}$ and $n_{d}$, respectively. Note that, in all cases, the matrix fluid is Newtonian; therefore, the respective systems are referred to as “Power–law–Newtonian”, denoted as “*PL–N*”. In the distinct case of $n_{d}=1$, the respective system is referred to as “Newtonian–Newtonian”, denoted as “*N–N*”.

Using the imposed shear rate magnitude, ${\dot{\gamma }}_{0}=2U/H$, and the single phase power–law formulation, $\eta_{d}=k_{d}{\dot{\gamma }}_{0}^{n_{d}-1}$, the so-called equivalent viscosity ratio of a Newtonian–Newtonian system is calculated as:

(27) $$\lambda_{eq}=\lambda_{PL}{\dot{\gamma }}_{0}^{n_{d}-1},$$

which is correlated to *N*–*N* system: a Newtonian–Newtonian with characteristic parameter λ to a Power–law–Newtonian with characteristic parameter $n_{d}$. This correlation is possible because, in both systems, the stresses exerted on the surface of the droplet depend on the term $\eta_{c}{\dot{\gamma }}_{0}$.

In our simulations, the shear rate magnitude is much lower than unity, i.e., ${\dot{\gamma }}_{0}=5\times 10^{-5}$. Hence, the aforementioned correlation leads to two most important assumptions:

1. shear-thinning droplets, i.e., $n_{d}<1$, would behave like higher-viscous ratio Newtonian droplets, i.e., λ>1;
2. shear-thickening droplets, i.e., $n_{d}>1$, would behave like lower-viscous ratio Newtonian droplets, i.e., λ<1.

**Figure 5:**
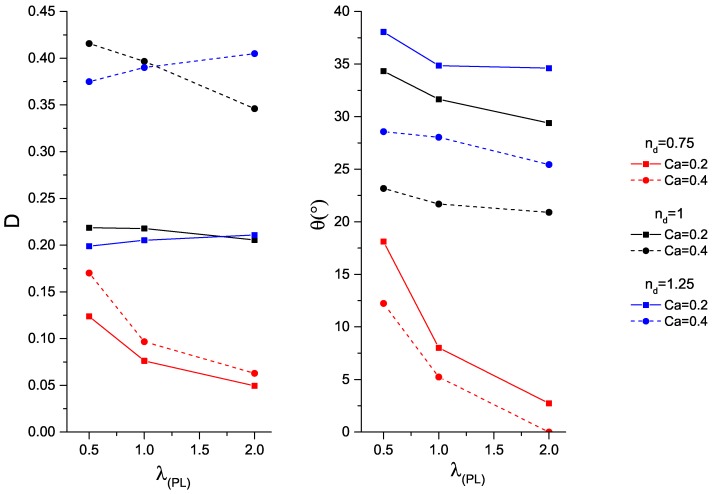
(**Left**) droplet deformation; and (**right**) orientation angle as a function of the power–law viscosity ratio, $\lambda_{PL}=k_{d}/\eta_{c}$, for power–law exponents $n_{d}=0.75,\;1,\;1.25$, with confinement ratio, 2R/H=0.4.

First, the impact of the power–law viscosity ratio is examined, i.e., $\lambda_{PL}=0.5,1,2$, at Ca=0.2,0.4, and $n_{d}=0.75,1,1.25$. The deformation parameters and orientation angles are plotted in Figure 5, which is based on 2D simulation results (all the other simulations are 3D). When Ca increases from 0.2 to 0.4, the droplet deforms to a greater extent and orients more to the direction of the flow. In comparison with the Newtonian droplet, shear-thinning droplets deform less, and shear-thickening droplets exhibit an opposite deformation trend. However, both Newtonian and shear-thickening droplets orient more to the direction of the flow with increasing $\lambda_{PL}$. Moreover, shear-thinning droplets show the lowest orientation angles. This finding confirms that high-viscous droplets align more to direction of the flow.

**Figure 6:**
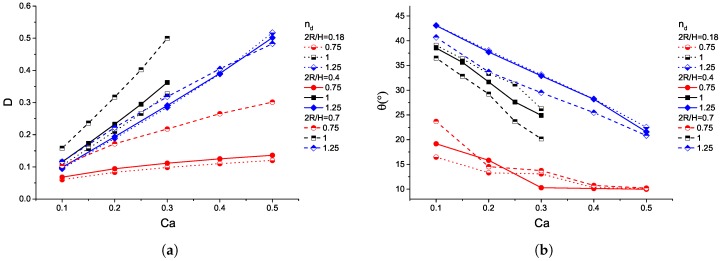
(**a**) droplet deformation and (**b**) orientation angle as a function of the capillary number, Ca, for exponents $n_{d}=0.75,\;1,\;1.25$, in various confinement ratios, 2R/H.

The behaviour of power–law droplets for $n_{d}=0.75,1,1.25$ is studied with $\lambda_{PL}=1$, and 2R/H=0.18,0.4,0.7 (see Figure 6a,b). Thus, these simulations compare a shear-thinning, a Newtonian and a shear-thickening droplet, i.e., $n_{d}=0.75,1,1.25$ in very weak, moderate and highly confined cases, i.e., 2R/H=0.18,0.4 and 0.7, respectively, where the capillary numbers range from 0.1 to 0.5. At Ca=0.5, the correlations between power–law exponents and equivalent viscosity ratios are

- shear-thinning: $n_{d}=0.75\to \lambda_{eq}\approx 13$,
- shear-thickening: $n_{d}=1.25\to \lambda_{eq}\approx 0.08$.

The critical capillary numbers in *N–N* systems of such viscosity ratios exceed greatly the value of 0.5, as reported in [28], and, therefore, no breakups are expected. However, some droplets deviate from the ellipsoid shape, e.g., for the case of $n_{d}=1.25$.

Figure 6a,b show the deformation parameters and the orientation angles as a function of Ca. The droplets deform to higher extents and turn more to the orientation of the flow with increasing capillary numbers and confinements. In particular, the Newtonian droplets present the biggest deformation, and their orientation angle trend lines lie between those for $n_{d}=0.75$ and 1.25. Furthermore, droplets with $n_{d}=0.75$ show the smaller values of both deformation and orientation angles, while droplets with $n_{d}=1.25$ depict the highest orientation angle values, even though they deform less than the Newtonian ones.

The deformation parameter trend lines indicate that the wall approximation has a weak influence on the deformation up to the confinement 2R/H=0.4. More specifically, the increase of confinement ratio from 2R/H=0.18 to 0.4 affects the droplets with $n_{d}=0.75$ mildly, and the ones with $n_{d}=1.25$ even more insignificantly. However, upon further increase to 2R/H=0.7, the droplets with $n_{d}=0.75$ deform considerably more. The specific droplets are subjected to high built-up stresses on the surface due to a lack of further tumbling, as indicated from the orientation angle values, in a similar mechanism for the highly-viscous Newtonian droplets [4]. On the other hand, the droplets with $n_{d}=1.25$ intensify their tumbling, i.e., decreasing θ in Figure 6, hence the built-up stresses have a moderate impact on the deformation. This behaviour is also illustrated by a low-viscous Newtonian droplet. Interestingly, in the case of Ca=0.5, increasing confinement of droplets with $n_{d}=1.25$ leads to smaller deformations. This can be linked to the finding that increasing confinement suppresses breakup for low viscosity Newtonian droplets [28,30].

**Figure 7:**
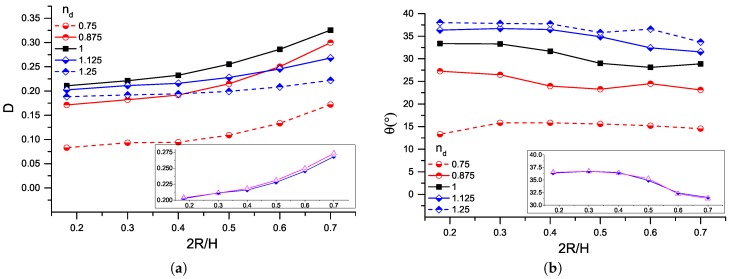
(**a**) droplet deformation and (**b**) orientation angle as a function of the confinement ratio, 2R/H, for various power–law exponents, $n_{d}$, at Ca=0.2. Insets: comparison of droplet characteristics for nd=1.125 and the Newtonian droplet of λ=0.3 (magenta-triangle).

Figure 7 shows the deformation and the orientation angle as a function of the confinement ratio. The insets compare the results from the *PL–N*/$n_{d}=1.125$ and the *N–N*/λ=0.3 systems. In general, increasing confinement ratios lead to increasing deformation parameters and decreasing orientation angles. Similar to the findings in Figure 5, the Newtonian droplet deforms the most, while it turns less than the shear-thinning droplets and more than the shear-thickening ones. More interestingly, power–law droplets with different exponents behave similarly. For example, shear-thinning droplets with $n_{d}=0.75$ and $n_{d}=0.875$ show a moderate increase of deformation rate when 2R/H increases to 0.4 and a more significant increase afterwards. In addition, their orientation is weakly affected by the presence of the walls, as seen in Figure 7. Meanwhile, the deformation of shear-thickening droplets present considerably less variations.

Finally, we consider equivalent systems including the *N–N*/λ=0.3 and *PL–N*/$n_{d}=1.125$ systems, where the capillary number, Ca, is 0.5, and the confinement ratio 2R/H=0.37. Notice that the Newtonian droplet undergoes a binary breakup in these conditions (see Figure 4). To achieve an equivalent viscosity ratio $\lambda_{eq}=0.3$, $n_{d}\approx 1.2747$. Therefore, the system *PL–N*/$n_{d}=1.2747$ is also included in this comparison. Hence, three systems are evaluated at Ca=0.5, 2R/H=0.37.

**Figure 8:**
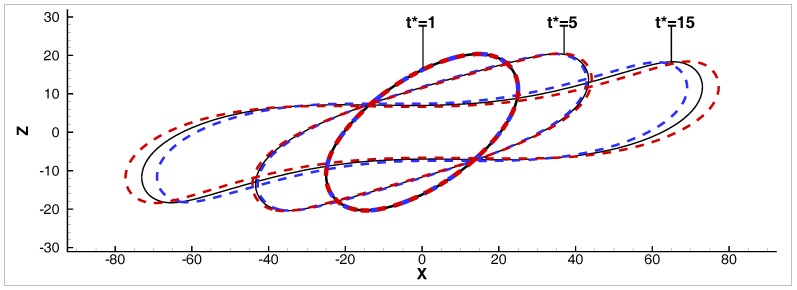
Transient droplet shapes, in the *x*-*z* middle plane, in systems *N–N*/λ=0.3 (solid-black), *PL–N*/$n_{d}=1.125$ (dashed-blue), and *PL–N*/$n_{d}\approx$1.2747 (dashed-red lines), at time instants $t^{*}$ = 1, 5, 15.

Figure 8 shows the shape evolution of the droplets at three time instants: $t^{*}=1,\;5,\;15$, where $t^{*}={\dot{\gamma }}_{0}t$ and *t* is the time step. The droplets deform to an identical ellipsoidal shape first, which is similar to a small deformation in a steady state. At a later time instant, i.e., $t^{*}=5$, the droplet shapes are slightly different and they deviate from ellipsoidal. Specifically, in *PL–N*/$n_{d}=1.125$, the droplet has elongated less compared to the one in *N–N*/λ=0.3, while the opposite is observed in *PL–N*/$n_{d}\approx$1.2747. Finally, at $t^{*}=15$, a long neck and differently oriented curvatures have been formed in all systems, where the droplet shapes differ considerably more than those in time instant $t^{*}=5$.

**Figure 9:**
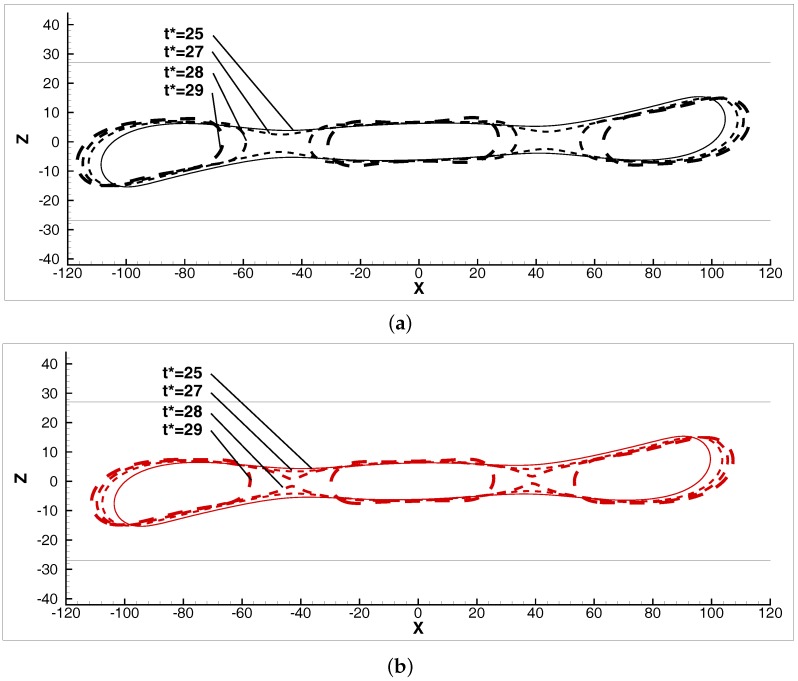
Transient droplet shapes close to the critical breakup time, in the *x*-*z* middle plane, in systems (**a**) *N–N*/λ=3 and (**b**) *PL–N*/$n_{d}\approx 0.8837$.

Figure 9 illustrates the shape evolution of the droplets close to breakup at Ca=0.5, 2R/H=0.7. Contrary to the previous observations regarding the low-viscous and shear-thinning droplets, the elongation does not cease due to the effects from the high approximation of the walls. Additionally, two regions between the centre and the two edges of the drop become very thin at $t^{*}=27$. These regions produce two pinch-off points at later times, leading to ternary breakups.

## 4. Conclusions

Dynamical behaviour of droplets under simple shear has been systematically studied under a broad range of confinement and viscosity ratios. Depending on viscosity ratios, confinement ratios have different effects on the critical capillary number: as the confinement increases, the critical capillary number increases for λ<1 but decreases for λ>1, and, for λ=1, the critical capillary number is kept at a value of around 0.4, regardless of the confinement ratio.

For Newtonian droplets in power–law matrix fluid, increasing confinement leads to an increase in droplet deformation for a wide range of viscosity ratios. The orientation angle of the deformed droplet decreases with increasing confinement or viscosity ratio. Meanwhile, the shear-thinning droplets behave similarly to highly-viscous Newtonian droplets, which are affected more significantly by confinement when compared to the shear-thickening.

The present work is the first step towards understanding how simple power–law fluids will respond to a shear flow in confinement. Experimental validation and extension to more realistic microfluidic geometries will be required to make the conclusions more useful for design of microfluidic devices. The code we used has been validated previously for Newtonian fluids in complex flow geometries e.g., [18]. As most available experimental data deal with viscoelastic fluids, we would be keen to extend our work from power–law fluids to viscoelastic ones, which will be a major step for code development.
